# Factors affecting variations of soil pH in different horizons in hilly regions

**DOI:** 10.1371/journal.pone.0218563

**Published:** 2019-06-19

**Authors:** Yun-Yi Zhang, Wei Wu, Hongbin Liu

**Affiliations:** 1 College of Resources and Environment, Southwest University, Chongqing, China; 2 College of Computer and Information Science, Southwest University, Chongqing, China; Universidade de Lisboa Instituto Superior Tecnico, PORTUGAL

## Abstract

Soil pH is a key factor that controls soil nutrient availability, soil microbial activities, and crop growth and development. However, studies on the soil pH variations of cultivated lands in different horizons at the regional scale remain limited. In this work, 348 soil samples were collected from three soil horizons (A, B, and C) at 120 sites over the hilly region of Chongqing, southwestern China. Six topographic indicators, four climate parameters, and parent material were considered. Classification and regression trees (CARTs) were applied to investigate the relationships between soil pH and the variables in the A, B, and C horizons. Model performances were evaluated by root mean square error (RMSE), relative root mean square error (RRMSE), and coefficient of determination (R^2^). Results showed that soil pH increased obviously from the A to C horizons. Soil pH was predicted well by the forcing factors with the CART models in all horizons. RMSE, RRMSE, and R^2^ varied between 0.37 and 0.435, between 5.93 and 7.23%, and between 0.71 and 0.80, respectively. The relative importance of the studied variables to soil pH differed with the horizons. Annual temperature range (ATR), terrain wetness index (TWI), and Melton ruggedness number were critical factors that controlled soil pH variability in the A horizon. Parent material, precipitation of warmest quarter (PWQ), ATR, and TWI were important variables in the B horizon. Parent material, PWQ, ATR, and precipitation were key factors in the C horizon. The results are expected to provide valuable information for designing appropriate measurements for agricultural practices and preventing soil acidification.

## Introduction

Soil properties are closely associated with soil-forming/environmental forcing factors, such as topography, climate, and parent material [1]. The relationship between soil properties and soil-forming factors is an issue that has been studied all over the world [2–7]. Soil pH is a measurement of soil acidity and alkalinity [8], thereby representing the H^+^ concentration in the soil solution. Soil pH is a key index of soil properties, which was considered as one of the main variables influencing other soil properties [9]. Studies have shown that soil pH can influence crop yields, soil nutrient release, and soil microbial activity to a large extent [10, 11]. If farmland soil is too acidic or too alkaline, then land production will be limited [12].

Soil pH is an important regulator of soil and is inevitably controlled by different soil-forming factors [13–16]. Previous studies have reported that factors associated with the variations in soil pH differ with locations and scales [13, 15, 17–19]. At the global scale, soils collected from different climates have distinct soil pH. Soils from arid climates are commonly alkaline with a high soil pH. By contrast, soils from humid climates are commonly acidic with a low soil pH [20]. Precipitation and potential evapotranspiration control soil pH variations at the global scale [13]. Additionally, the effects of climate factors on soil pH variations are observed at regional scales [20–22]. For example, Cheng et al. [21] reported that soil pH has an obviously negative correlation with mean temperature and mean precipitation. Chytrý et al. [22] also found that soil pH presents a downward trend when precipitation increases. Meanwhile, the relationships between soil pH and terrain indicators are site-dependent. For example, Moore et al. [17] found that slope and the topographic wetness index (TWI) significantly influenced the soil pH in an agricultural landscape in Colorado. Chen et al. [19] found that topographic aspect and slope were the main factors that affected soil pH in a mountainous area in southern Taiwan. Li et al. [15] reported that location-specific terrain features and catchment-related hydrological activities can influence regional soil pH. Others also reported that soil pH variations are influenced by parent materials. For example, soils developed from Triassic sandstones and Quaternary sands have significantly different pH values [23]. Reuter et al. [24] reported that the spatial distribution of soil pH is highly dependent on the nature of the parent material. Fabian et al. [16] found low pH values on top of crystalline bedrocks and high pH values over areas underlain by limestone. However, studies on the effects of environmental forcing factors on soil pH in sub-soils remain limited.

A lot of statistical models have been applied to investigate the relationships between the environmental forcing factors and soil pH [25–28]. Among them, the classification and regression tree (CART) is a non-parametric decision tree algorithm that is easy to build and explain [29]. CART could help users make a decision among several choices. CART does not need any model assumptions and could automatically address categorical and continuous variables. Thus, it has been widely applied to explore non-linear relationships between independent and dependent variables [29–31]. One interesting outcome of CART is the relative importance of independent predictors to the target. Furthermore, cross-validation complemented with CART could effectively avoid overfitting [29].

In consideration of the advantages of CART and the lack of studies on the impact of environmental forcing factors on soil pH in sub-soils, we attempted to (1) examine the spatial variability of soil pH in different horizons and (2) investigate the relationships between environmental forcing factors (climate, parent material, and topography) and soil pH in surface and sub-soils. The work was conducted in a hilly region of southwestern China.

## Materials and methods

### Study area

**Fig 1:**
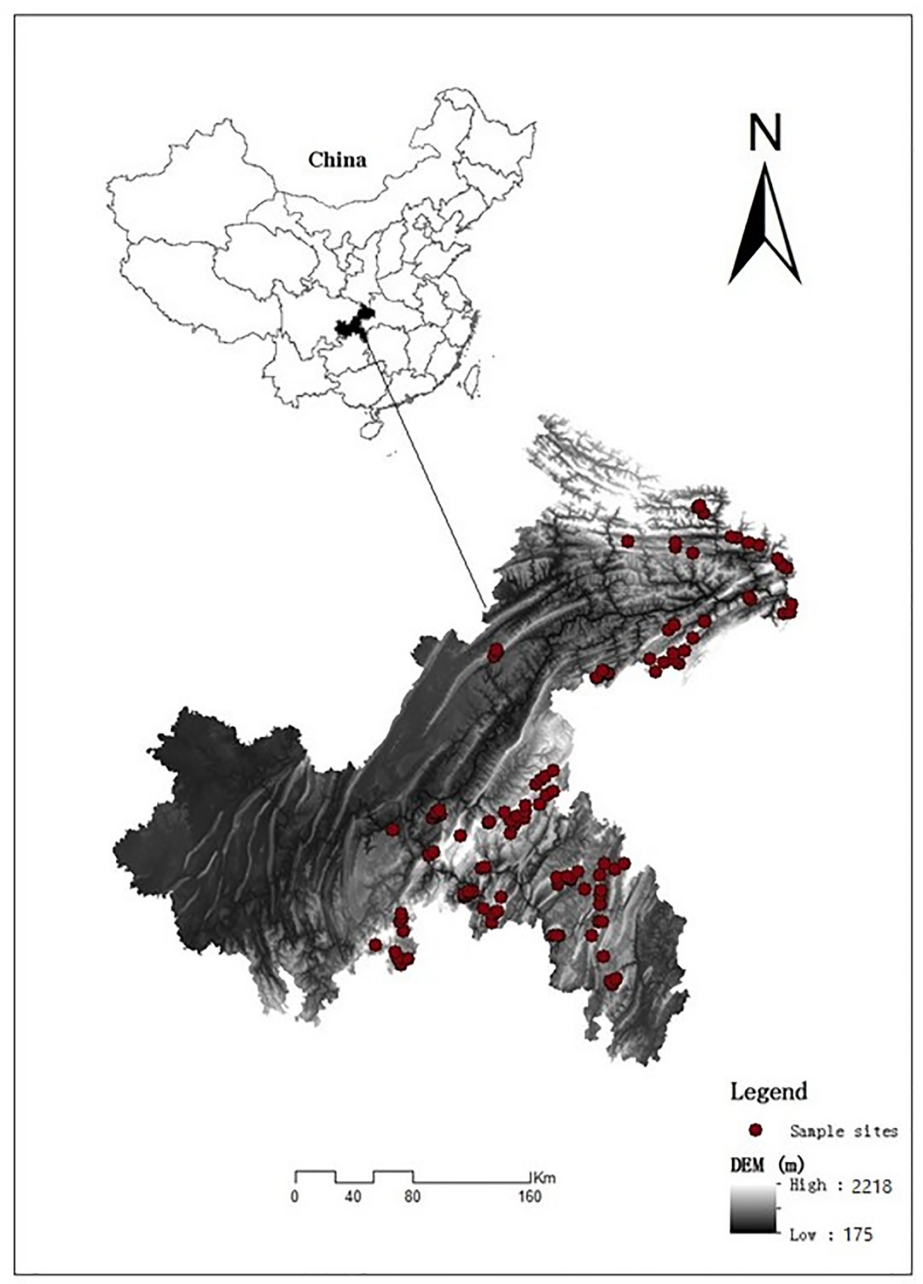
Maps of study area location and sample sites.

The study area (105°17′ to 110°11′E, 28°10′ to 32°13′N) is located in southwestern China (Fig 1). The length between east and west is 470 km, and the breadth between south and north is 450 km. The elevation varies between 175 and 2218 m, and the slope is between 0.45° and 42°. The climate is subtropical monsoon humid climate with a mean annual temperate of 13.35°C and mean annual precipitation of 1317 mm. The parent materials are Silurian marlite, Triassic limestone, Permian limestone, Ordovician limestone, Cambrian limestone, and Jurassic limestone.

### Data

The study was conducted in the hilly region of the study area, where tobacco is planted in April and harvested in August. A total of 120 soil profile samples were collected from the upland in September–November 2014 and 2015. The study did not involve any protected area, private land, endangered or protected species. And no specific permissions were required for these locations/activity. Up to three layers (A: surface soil layer, B: subsoil layer, C: substrate layer) were identified for each profile. At each site, a pit with 1 m (width) × 2 m (length) × 1.5 m (height) was dug by local farmers. For each horizon, 500 g of thoroughly mixed soil was used for chemical analyses at each site. Finally, 119, 119, and 110 soil samples were used for the A, B, and C layers, respectively. All soil samples were air-dried and passed through a 2 mm soil sieve prior to data analyses. Soil pH was determined in a soil-to-water suspension of ratio of 1:2.5 (m/v) with a glass electrode.

Soil-forming factors, namely, topography, climate, and parent material, were considered in this work. Given that the cultivation system and crop species at each site were similar, the organisms that were also a soil-forming factor were not used in this study. By reviewing published papers and many previous experiments, six topographic variables and four climate parameters were used (Table 1). The topographic variables were channel network, valley depth, Melton ruggedness number (MNR), elevation, slope, and TWI; they were derived from a 90 m×90 m grid Digital Elevation Model (DEM) by the software System for Automated Geoscientific Analyses (2014) V.2.2.7. The climate parameters were annual mean temperature, annual precipitation, precipitation of warmest quarter (PWQ), and annual temperature range (ATR) between the warmest and coldest month (Table 1). They were derived from the WorldClim Database. Parent material is a vital factor for soil pH, as noted during the sampling.

**Table 1:** Description of the used topographic indicators and climate variables.

	Factor	Unit	Description
**Topography**	Elevation	m	The height of a location above the Earth’s sea level
	Slope	°	The local hill slope gradient
	TWI	Dimensionless	An index that can quantify the control of local topography in hydrological processes and indicate the spatial distribution of soil moisture and surface saturation
	Valley Depth	m	Vertical distance to a channel network base level
	Channel Network	Dimensionless	A network of potential water flow paths
	MNR	Dimensionless	Melton ruggedness number. A simple flow accumulation related index
**Climate**	ATR	°C	Annual temperature range between the warmest and coldest month
	PWQ	mm	Precipitation of warmest quarter
	Precipitation	mm	Mean annual precipitation
	Temperature	°C	Mean annual temperature

### Modeling

Classification and regression tree (CART) is a non-parametric decision method that was proposed by Breiman et al. [29]. The model follows the recursive partitioning rules to generate a classification (categorical) or regression (numeric) tree depending on the response variable. Regression tree was used in the present work. This technique does not need any assumption on model and data distribution.

One outcome of the CART models is the relative importance of each variable to the system [32–35]. In the present study, CART was applied to investigate the relative importance of the factors that control the variations in soil pH in different horizons. The following equation was used to obtain the relative importance of variable x_j_ [29]:

$${RI}_{j}=\sum_{k}\Delta s(j,k),$$ (1)

where Δs(j,k) is the reduction in the mean squared error S if node k were split by variable x_j_.

The appropriate parameters for CART were obtained after several experiments. The minimum numbers of child nodes and parent nodes were 1 and 2, respectively. The maximum tree depth was 5. Tenfold cross-validation was used in the present study to avoid overfitting. Detailed information about CART may be found in Breiman et al. [29].

In assessing the accuracy of the model, three indices, namely, root mean square error (RMSE), relative root mean square error (RRMSE), and coefficient of determination (R^2^), were used in the current study.

$$RMSE=\sqrt{\frac{\sum_{i=1}^{n}{\left(y_{i}-{\hat{y}}_{i}\right)}^{2}}{n}},$$ (2)

$$RRMSE=\frac{RMSE}{\bar{y}},$$ (3)

$$R^{2}=1-\frac{\sum_{i=1}^{n}{\left(y_{i}-\bar{y}\right)}^{2}}{\sum_{i=1}^{n}{\left(y_{i}-\hat{y_{i}}\right)}^{2}},$$ (4)

where y and ŷ denote the observed and predicted soil pH, respectively'; and ȳ is the mean value of the observations. According to [36], model accuracy is considered excellent when RRMSE < = 10%, good if 10% <RRMSE< = 20%, fair if 20% <RRMSE< = 30%, and poor if RRMSE >30%. The models with the lowest values of RMSE and highest values of R^2^ showed superior performance.

### Statistical analysis

Pearson correlation coefficients were calculated to determine the correlations between the soil pH and soil-forming factors in different horizons and the correlations among soil-forming factors. A one-way analysis of variance (ANOVA) with Games Howell was applied to test the differences in soil pH among the parent materials and horizons. The statistical analyses were conducted using Microsoft Excel 2010 and SPSS V.22.

## Results

### Descriptive statistics

The descriptive statistics of soil pH in different horizons, climate factors, and terrain indicators are listed in Table 2. The minimum and mean values of soil pH increased from the A to C horizons. The values of the coefficient of variation (CV) of pH indicate the low variability of soil pH at each horizon over the area (CV<25%).Among the terrain indicators, elevation had low variability, MNR had high variability (CV% >75%), and others had medium (CV% = 25%–75%) variabilities over the site. Among the climate indicators, the CVs showed that temperature, ATR, and PWQ had low variability and that precipitation had medium variability across the area.

**Table 2:** Descriptive statistics of soil pH in A, B, and C horizons; topographic indicators; and climate variables.

		Min	Max	Mean	SD	CV(%)	Skewness		Kurtosis
**pH**	A horizon	3.94	7.74	5.34	0.83	15.54	1	0.36	
	B horizon	4.27	8.02	6.02	0.82	13.62	0.16	-0.35	
	C horizon	4.47	7.81	6.31	0.77	12.25	-0.21	-0.37	
**Topography**	Elevation(m)	569	1569	1132.7	254.13	22.44	-0.37	-0.82	
	Slope (°)	0.45	42.01	12.73	8.01	62.92	1	1.26	
	TWI	5.11	20.33	8.30	3.45	41.57	2.06	3.64	
	Valley Depth (m)	0	884.07	212.1	145.24	68.48	1.01	2.62	
	Channel Network	428.14	1603.14	950.77	273.57	28.77	0.47	-0.66	
	MNR	0	17.23	2.75	2.93	106.55	2.01	5.48	
**Climate**	ATR (°C)	26.20	30.20	28.08	0.94	3.35	0.74	-0.19	
	PWQ (mm)	490	581	536	24.53	4.58	-0.07	-1.15	
	Precipitation (mm)	1170	1429	1316	67.14	5.1	-0.31	-1.1	
	Temperature (°C)	9.90	16.80	13.36	1.53	11.45	0.23	-0.57	

Min = minimum, Max = maximum, SD = standard deviation, CV = coefficient of variation.

### ANOVA

**Fig 2:**
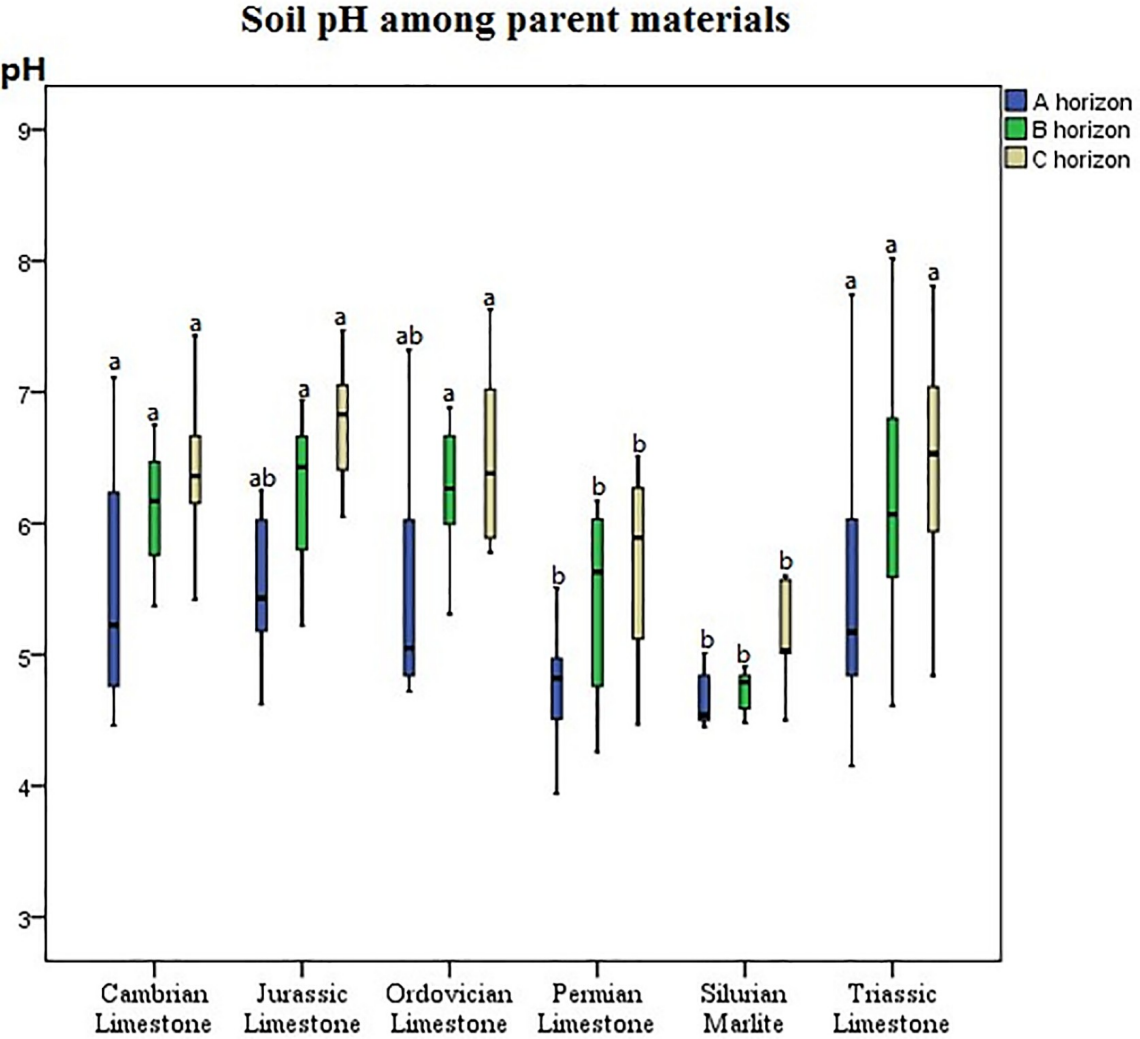
Differences in soil pH among parent materials in the horizons. Values with the same letter indicate no significant difference (p < 0.05).

The differences in the soil pH among the parent materials in the horizons were tested by ANOVA, and the results are shown in Fig 2. Obviously, soil pH increased from the A to C horizons for each parent material. Furthermore, significant differences in soil pH were observed among the parent materials. Soils developed from Cambrian, Jurassic, Ordovician, and Triassic limestones had higher soil pH than did those from Permian limestone and Silurian marlite for each horizon.

### Correlation analysis

The Pearson correlations between soil pH and soil-forming factors are listed in Table 3. From the A to C horizons, soil pH was positively significantly correlated with ATR and valley depth. The correlation between the used factors is presented in Table 4. Elevation and channel network were significantly correlated with PWQ, precipitation, and temperature. ATR was positively correlated with valley depth.

**Table 3:** Pearson correlation between pH and topographic indicators and climate variables.

	Topography						Climate			
	Elevation (m)	Slope (°)	TWI	Valley Depth (m)	Channel Network	MNR	ATR (°C)	PWQ (mm)	Precipitation (mm)	Temperature (°C)
**A**	-0.014	0.137	-0.005	0.315**	0.570	0.062	0.365**	0.111	-0.038	-0.073
**B**	-0.046	0.019	0.029	0.268**	-0.040	-0.043	0.393**	0.020	-0.160	-0.011
**C**	-0.089	-0.036	0.048	0.212**	-0.042	-0.056	0.435**	0.076	-0.112	0.026

**Significance level at p<0.01.

**Table 4:** Correlation between used factors.

	Topography						Climate			
	Elevation(m)	Slope(°)	TWI	Valley Depth(m)	Channel Network	MNR	ATR (°C)	PWQ (mm)	Precipitation (mm)	Temperature(°C)
**Elevation**	1									
**Slope**	0.008	1									
**TWI**	-0.016	-0.479**	1							
**Valley Depth**	-0.200*	0.097	0.107	1						
**Channel Network**	0.733**	-0.167	0.302**	0.082	1					
**MNR**	0.03	0.514**	0.134	0.218*	0.036	1				
**ATR**	0.1	0.136	-0.094	0.382**	0.12	-0.042	1			
**PWQ**	0.443**	0.096	-0.028	-0.076	0.410**	0.056	0.465**	1		
**Precipitation**	0.411**	-0.005	0.065	-0.233*	0.465**	0.036	0.067	0.867**	1	
**Temperature**	-0.942**	-0.019	-0.075	-0.009	-0.794**	-0.096	-0.234*	-0.491**	-0.413**	1

*Significance level at p<0.05.

**Significance level at p<0.01.

### CART models

**Fig 3:**
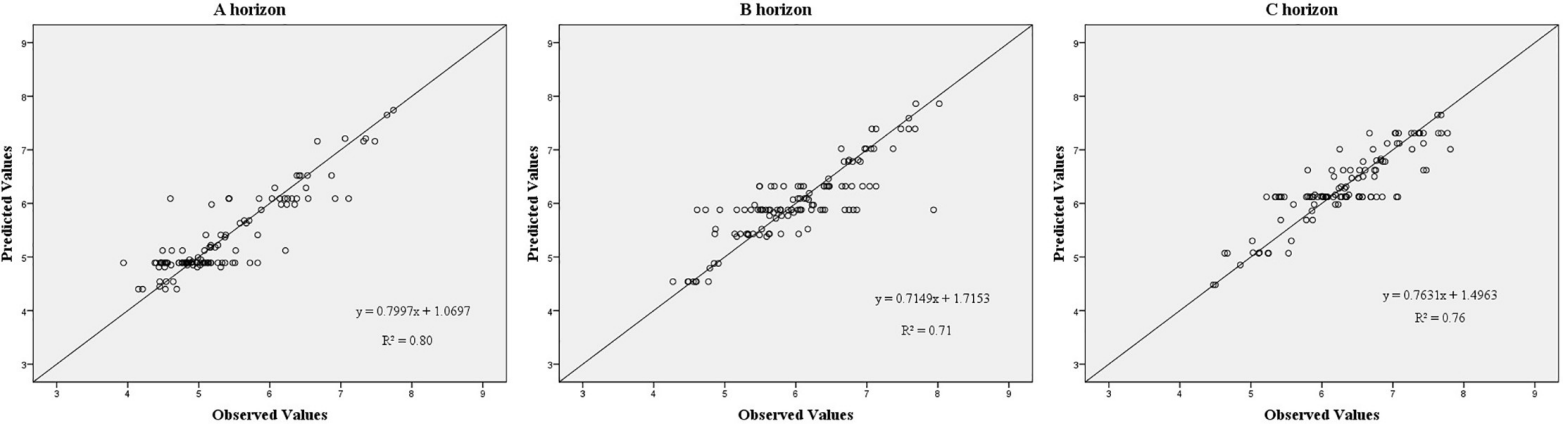
Scatter plots of observed and predicted soil pH based on CARTs for each horizon: a) A horizon, b) B horizon, and c) C horizon.

Three trees were constructed to explore the relationships between the used soil-forming factors and the soil pH for the three horizons. The values of RMSE, RRMSE, and R^2^ are shown in Table 5. The relationship between observed and predicted pH values is shown in Fig 3.

**Table 5:** Accuracy assessment indices of CART models in different horizons.

Horizon	RMSE	R^2^	RRMSE (%)
**A**	0.37	0.80**	6.94
**B**	0.435	0.71**	7.23
**C**	0.374	0.76**	5.93

** Significance level at p<0.01.

RRMSE indicates that the models were excellent for the three horizons. The models could explain 80%, 71%, and 76% of the total variations in soil pH for the A, B, and C, horizons, respectively. In terms of RMSE and R^2^, the models provided the best estimations of soil pH for the A horizon and the worst estimations for the B horizon.

**Fig 4:**
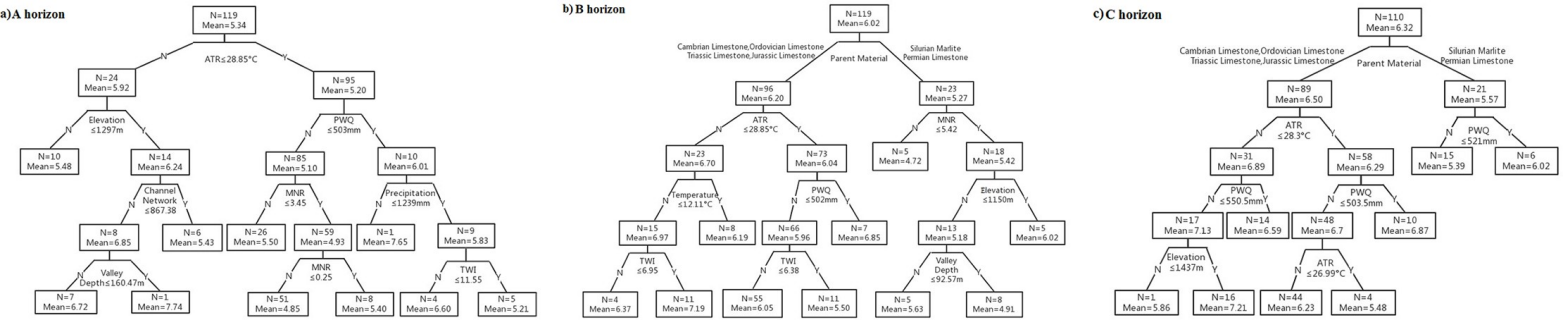
CART analysis for each horizon: a) A horizon, b) B horizon, c) C horizon.

Three binary trees were created by the CART algorithm (Fig 4). For the A horizon, ATR was the first splitting rule (Fig 4A). The samples with low ATR (≤28.85°C) had lower soil pH than did those with high ATR (>28.85°C). Then, the samples were split by elevation and PWQ for the left and right nodes, respectively. For the left sub-tree, the samples at low areas (elevation < = 1297 m) had high pH values. These samples were further split by channel network and valley depth. For the right sub-tree of the root, the samples with low pH values were identified by PWQ less than or equal to 503 mm. These samples were then split by MNR. The samples with low pH values were identified with MNR < = 3.45. For the B horizon (Fig 4B), parent material is the most powerful descriptor to split the samples. The samples collected from Permian limestone and Silurian marlite had lower soil pH than did the samples from other parent materials. For the left sub-tree, the samples with low soil pH values were identified by ATR < = 28.85°C. They were further split by PWQ (502 mm) and TWI (6.38). The samples with a mean pH of 6.05 were found in the areas with PWQ > 502 mm and TWI > 6.38. For the right sub-tree of the root, the samples were split by MNR. The samples with high values of soil pH were identified with MNR < = 5.42, and they were further split by elevation and valley depth. For the C horizon, the samples were also first split by parent material (Fig 4C). The samples collected from Cambrian, Jurassic, Ordovician, Triassic, and Jurassic limestone had higher soil pH than did the samples from Silurian marlite and Permian limestone. This condition was the same as that for the B horizon. In level 2, ATR, PWQ, and elevation were used to divide the samples of the left sub-tree. The samples with mean soil pH of 6.23 were found in the area with PWQ higher than 503.5 mm and ATR between 26.99°C and 28.3°C. Additionally, PWQ was applied to split the right sub-tree. The samples had lower values of soil pH in the areas with PWQ < = 521 mm.

### Relative importance of soil-forming factors

**Fig 5:**
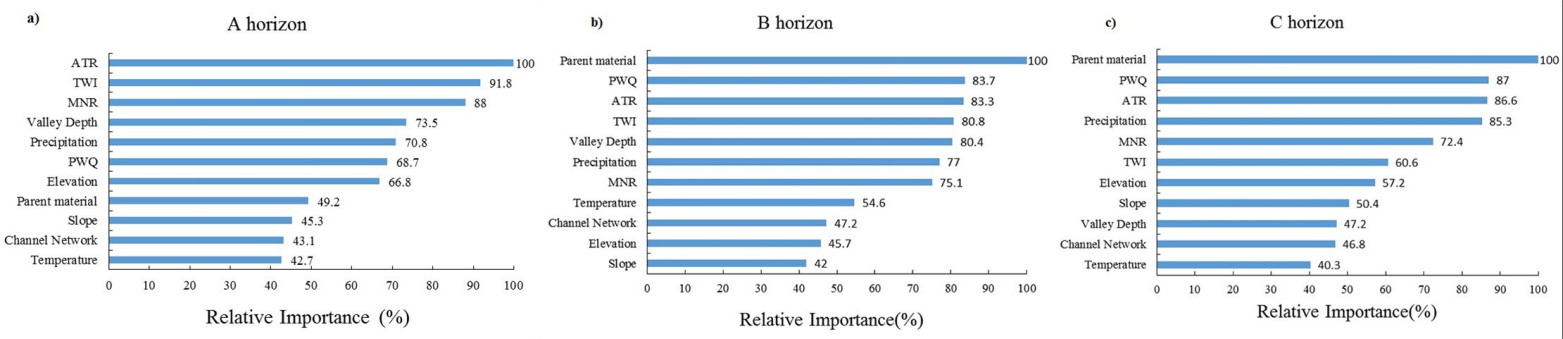
Variable importance rankings produced by CART models for each horizon: a) A horizon, b) B horizon, c) C horizon.

Relative importance is one of the interesting outputs produced by CART models. The relative importance of soil-forming variables to soil pH in different horizons is presented in Fig 5. The variables provided different levels of relative importance for different horizons. For the A horizon, the most important factors affecting soil pH variation were ATR, TWI, and MNR (Fig 5A). These variables had relative importance values that exceeded 80%. For the B horizon, parent material, PWQ, ATR, TWI, and valley depth had high relative importance values greater than 80% (Fig 5B). For the C horizon, most of them (parent material, PWQ, ATR and precipitation) also had high values of relative importance (>85%) to soil pH variation (Fig 5C). On average, the rank order of the factors was climate > topography > parent material for A horizon and parent material > climate > topography for B and C horizons. Obviously, the effects of parent material and precipitation-related factors (precipitation and PWQ) on the soil pH variations significantly enhanced in deep layers.

## Discussion

The results of our study indicate that the relationships between soil pH and soil-forming factors varied with horizons.

In the A horizon, climate and topography mainly influenced soil pH variation. Many studies indicated that temperature and precipitation are important factors that control soil pH [21, 37, 38]. Temperature mainly influences rock weathering rate, and precipitation mostly affects material flow. To a certain extent, climate can affect the process of soil chemical reaction and thereby influence soil pH. Soils from different climates have distinct soil pH. The soils from arid climates are commonly alkaline with high soil pH. By contrast, soils from humid climates are commonly acidic with low soil pH [20]. Changing climate can influence regional rainfall to a certain degree. Increased precipitation can leach many alkaline basic cations from the topsoil, and this condition could lead to topsoil acidification. Chytrý et al. [22] reported a significant negative correlation between soil pH and the amount of precipitation possibly caused by the increased precipitation enhancing the leaching rate of some alkaline cations, such as Ca^2+^, Mg^2+^, K^+^, and Na^+^ along the terrain gradient. In some warm environments, the soil solution will contain a large amount of H^+^ ions because high temperatures can accelerate the accumulation of soil organic matter [39]. The dissociation of carbonic acid is also an important source of H^+^ in soil solution [40].

Topography affects soil pH mainly in two ways. One is by controlling water flow and material transport [17]. The other is by changing the local climate, the elevation can observably influence the local temperature and precipitation. In general, low temperature and abundant rainfall often occurs at high altitudes. Seibert et al. [14] found that topography and soil chemistry have a great correlation in the O-horizon, thereby indicating that the topsoil is strongly exposed to topographic controls. In the same study, they reported that topography factors derived from DEMs (Digital Elevation Models) are significantly correlated with soil pH. In the present region, two soil moisture-related indicators, TWI and MNR, were the most important topographic factors that affected soil pH. The TWI (ln(a/tan)), which combines local upslope contributing area and slope, is an important factor to quantify the topography control on hydrological processes [15, 18]. MNR is a simple flow accumulation related index and is associated with the regional catchment condition. Seibert et al. [14] reported that soil pH in the organic layer increases with the TWI. However, in our study, soil pH was slightly negatively correlated with the TWI (r = −0.005). This difference might be caused by the local special hilly topography [15].

ATR is a key factor in the A horizon. ATR was significantly positively correlated with soil pH in the three horizons (r = 0.365, 0.393, 0.435; p<0.01, for the A, B, and C horizons, respectively). Our study area is a typical hilly region covered with limestone. A high ATR will cause rocks to undergo continual thermal expansion. This process will increase rock weathering rate and increase the Ca^2+^ concentration in the soil solution, thereby causing an increase in soil pH. Here, ATR was significantly positively correlated with PWQ and valley depth (Table 4). Thus, high ATR is consistently accompanied by rich precipitation and a wavy terrain. Hence, our results showed that much Ca^2+^ was leached by precipitation along the terrain gradient; thus, the Ca^2+^ of the soil solution in the A horizon was low. This situation may explain why the soil pH in the A horizon was the lowest in the three horizons. PQW, a factor that involves temperature and precipitation proved to be an important variable in the B and C horizons. This was possibly because when high temperature expedites limestone weathering rate, the rich precipitation can significantly increase the leaching time. Therefore, more Ca^2+^ is lost in the A horizon and precipitated in the B and C horizons. This situation results in the higher values of soil pH in the B and C horizons than in the A horizon.

In the B and C horizons, parent material was the most essential factor that affected soil pH. Reuter et al. [24] reported that the distribution of soil pH is highly dependent on the nature of the parent material. In their study, low pH soils mainly developed from acid materials, and high pH soils mainly presented in the calcareous nature of the parent materials. Castrignano A et al. [41] also found that soils developed directly on carbonatic substrates, such as Leptosols and Regosols, show neutral or weakly alkaline pH, whereas those developed on glacial till (subject to brunification process, e.g., Cambisols; or to podsolization process, e.g., Podzols) have slightly to strongly acidic pH. Parent materials appear to have great importance for soil acidity; the pH of soils developed from Triassic sandstones is significantly higher than that from Quaternary sands [23]. In the present study, soils developed from Permian limestone and Silurian marlite obviously have lower soil pH than those from Cambrian, Jurassic, Ordovician, and Triassic limestone (Fig 2). High soil pH occurs at low altitudes where the processes of alkalinization and salinization raise the pH. Low soil pH is distributed on mountaintops because the production of granitic rocks can accelerate podsolization processes [42]. A similar result was obtained in the current study. Permian limestone and Silurian marlite were distributed at high altitudes with mean elevations of 1182 and 1201 m, respectively. Jurassic, Ordovician, and Triassic limestone were distributed at altitudes with mean elevations of 1016, 1038, and 1132 m, respectively. The low soil pH of Permian limestone and Silurian marlite might have been due to the low temperature restraining the decomposition of soil organic matter and thereby causing the accumulation of organic acid in soil.

Above 70% of the total variations in soil pH in the three horizons could be explained by the CART models. The uncertainty might be related to the lack of detailed farming practices, such as fertilization and drainage water quality, because human activities also had pronounced effects on soil properties [43]. Several variables, such as soil texture and soil porosity, were neglected in this study. The uncertainty of the method itself also existed. Given that the model only considered the relationship between the independent and dependent variables, the correlations among independent variables were not considered. The uncertainty of the A horizon was the lowest among the horizons in this study possibly because the soils in the A horizon were more exposed to the environment and were easily influenced by the studied factors.

## Conclusion

On the basis of the assumption that climate, topography, and parent material have varied influence on soil pH in different horizons, CART models were applied to investigate soil pH variability and the relationships between soil pH and the studied soil-forming factors in different horizons across a hilly region in Chongqing, southwestern China. The results indicated that the values of soil pH increased along the horizon gradient from the A to C horizons. Different soil-forming factors played different roles in the three horizons. In the A horizon, climate and topography had the greatest influence on soil pH. In the B and C horizons, the key factor that affected soil pH was parent material. Topography and climate also had great importance in the B horizon. In the C horizon, the importance of climate to soil pH variation increased, whereas that of topography decreased. These findings will be useful for understanding the relationships between soil-forming factors and soil pH in different layers and provide valuable information for designing suitable measurements for agricultural practices and preventing soil acidification.

## Supporting information

S1 File The data of A horizon. (XLSX)

S2 File The data of B horizon. (XLSX)

S3 File The data of C horizon. (XLSX)
